# Synthesis, crystal structure and charge-distribution validation of a new alluaudite-type phosphate, Na_2.22_Mn_0.87_In_1.68_(PO_4_)_3_

**DOI:** 10.1107/S2056989020010191

**Published:** 2020-07-31

**Authors:** Abdessalem Badri, Inmaculada Alvarez-Serrano, María Luisa López, Mongi Ben Amara

**Affiliations:** a Laboratory of Interfacial and Advanced Materials, Faculty of Sciences (FSM), University of Monastir, Monastir 5000, Tunisia; b Departamento de Química Inorgánica, Facultad de Ciencias Químicas, Universidad Complutense, 28040 Madrid, Spain

**Keywords:** crystal structure, indium phosphate, alluaudite structure type, disorder

## Abstract

Na_2.22_Mn_0.87_In_1.68_(PO_4)3_, sodium manganese indium tris­(phosphate) (2.22/0.87/1.68), was obtained in the form of single crystals by a flux method and was structurally characterized by single-crystal X-ray diffraction. The compound belongs to the alluaudite structure type (space group *C*2/*c*) with general formula *X*(2)*X*(1)*M*(1)*M*(2)_2_(PO_4_)_3_. The *X*(2) and *X*(1) sites are partially occupied by sodium [occupancy 0.7676 (17) and 1/2] while the *M*(1) and *M*(2) sites are fully occupied within a mixed distribution of sodium/manganese(II) and manganese(II)/indium, respectively. The three-dimensional anionic framework is built up on the basis of *M*(2)_2_O_10_ dimers that share opposite edges with *M*(1)O_6_ octa­hedra, thus forming infinite chains extending parallel to [10]. The linkage between these chains is ensured by PO_4_ tetra­hedra through common vertices. The three-dimensional network thus constructed delimits two types of hexa­gonal channels, resulting from the catenation of *M*(2)_2_O_10_ dimers, *M*(1)O_6_ octa­hedra and PO_4_ tetra­hedra through edge- and corner-sharing. The channels are occupied by Na^+^ cations with coordination numbers of seven and eight.

## Abstract 2

The title compound crystallizes in the alluaudite structure type. Its three-dimensional framework includes channels in which partially occupied sodium cations are situated.

## Chemical context   

The general structural formula of alluaudite-type phosphates is [*A*(2)*A*(2)][*A*(1)*A*(1)’*A*(1)’’_2_]*M*(1)*M*(2)_2_(PO_4_)_3_ (Hatert *et al.*, 2000 ▸); in the majority of natural alluaudites, the large crystallographic *A* sites are occupied by Na^+^, Ca^2+^ or Mn^2+^, and the distorted-octa­hedrally surrounded *M* sites are occupied by Mn^2+^, Fe^2+^, Fe^3+^, Al^3+^ or Mg^2+^ (Moore & Ito, 1979 ▸). Alluaudite-type phosphates are frequently used for practical applications such as corrosion inhibition, passivation of metal surfaces, or catalysis (Korzenski *et al.*, 1998 ▸; Kacimi *et al.*, 2005 ▸). Furthermore, as a result of the presence of channels, alluaudite-type compounds exhibit electronic and/or ionic conductivity properties (Warner *et al.*, 1994 ▸; Durio *et al.*, 2002 ▸). The possibility of inserting variable amounts of lithium into the channels of the alluaudite structure also makes the (Na_1–*x*_Li_*x*_)MnFe^3+^_2_(PO_4_)_3_ and (Na_1–*x*_Li_*x*_)_1.5_Mn_1.5_Fe^3+^_1.5_(PO_4_)_3_ compounds of value as potential battery materials (Hatert *et al.*, 2004 ▸; Trad *et al.*, 2018 ▸). A number of indium-bearing alluaudite-like compounds have also been synthesized, *i.e*. NaCdIn_2_(PO_4_)_3_ (Antenucci *et al.*, 1993 ▸), Na_3_In_2_(PO_4_)_3_ (Lii & Ye, 1997 ▸), and NaMn(Fe_1–*x*_In_*x*_)_2_(PO_4_)_3_ (Hatert *et al.*, 2003 ▸). In this paper, we report the structural study of a new alluaudite-type phosphate, Na_2.22_Mn_0.87_In_1.68_(PO_4_)_3_, which was obtained during our investigation of the Na_3_PO_4_–Mn_3_(PO_4_)_2_–InPO_4_ quasi system.

## Structural commentary   

**Figure 1:**
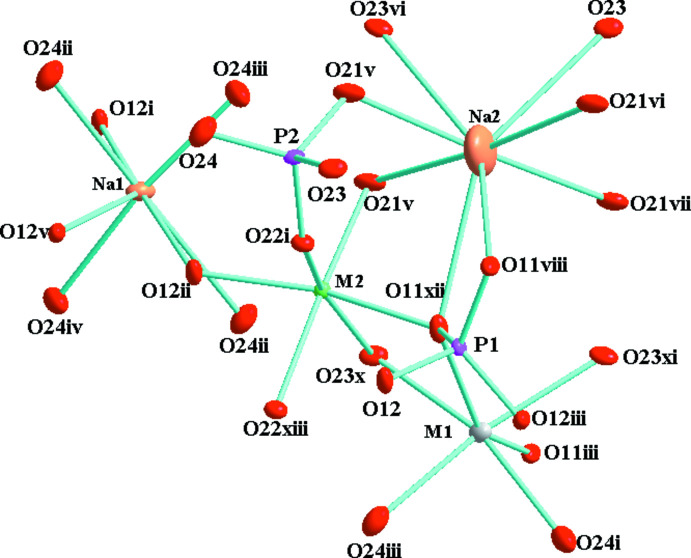
The principal building units of the alluaudite-type phosphate Na_2.22_Mn_0.87_In_1.68_(PO_4_)_3_ with displacement ellipsoids drawn at the 50% probability level. [Symmetry codes: (i) *x*, *y*, *z * − 1; (ii) *x* + 1, *y*, *z* + 1; (iii) *x* + 1, *y*, *z* + 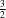; (iv) *x*, *y*, *z* − 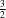; (v) *x*, *y*, *z* − 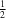; (vi) *x*, *y*, *z* + 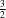; (vii) *x*, *y*, *z* + 2; (viii) *x* + 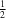, *y* − 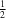, *z* + 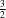; (ix) *x* − 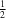, *y* − 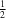, *z*; (*x*) *x* + 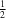, *y* + 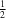, *z* − 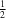; (xi) *x* + 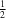, *y* + 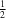, *z* + 1; (xii) *x* − 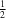, *y* + 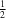, *z* − 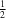; (xiii) *x* + 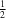, *y* + 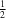, *z* + 2; (xiv) *x* + 1, *y*, *z*.]

**Figure 2:**
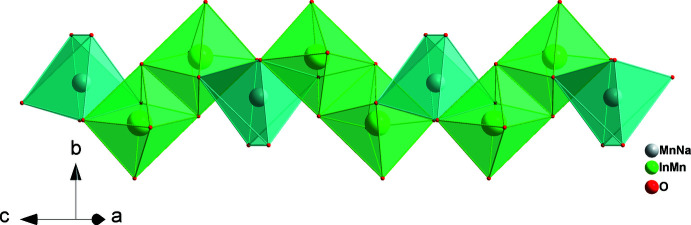
Infinite zigzag chain extending parallel to [10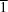], built of edge-sharing *M*(2)_2_O_10_ and *M*(1)O_6_ units.

**Figure 3:**
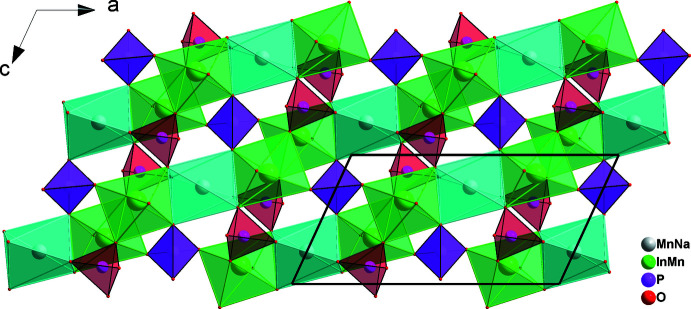
The connection of individual chains *via* PO_4_ tetra­hedra to give sheets perpendicular to [010]_._

**Figure 4:**
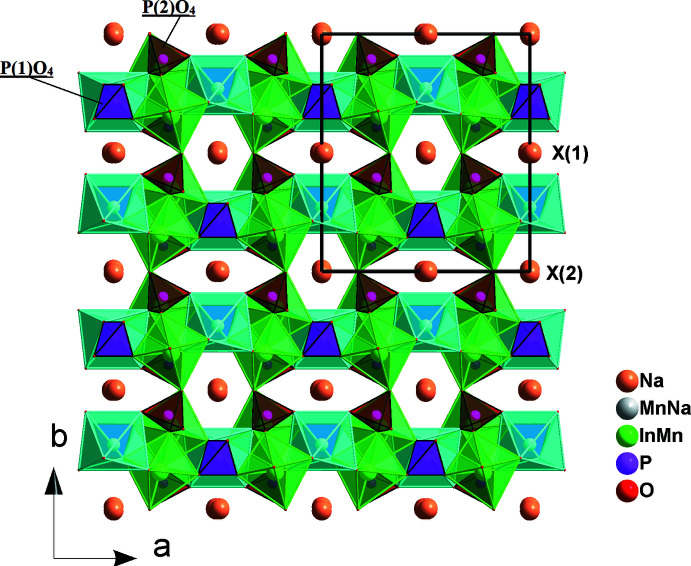
Projection of the Na_2.22_Mn_0.87_In_1.68_(PO_4_)_3_ structure along the [001] direction showing channels occupied by the Na^+^ cations.

The principal building units (Fig. 1 ▸) of the three-dimensional framework structure of Na_2.22_Mn_0.87_In_1.68_(PO_4_)_3_ are mixed-occupancy (Mn, Na) [= *M*(1); site symmetry 2] and (Mn1, In) [= *M*(2)] sites with distorted octa­hedral environments and two phosphate tetra­hedra (P1 and P2); the two sites associated with Na^+^ cations (Na1; Na2 with site symmetry 2) are partially occupied and are situated in the resulting voids. By edge-sharing, the (Mn,In)O_6_ octa­hedra form (Mn,In)_2_O_10_ dimers, which are linked by highly distorted (Mn,Na)O_6_ octa­hedra into infinite zigzag chains along [10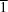] (Fig. 2 ▸). The connection of these chains through vertices belonging to P1O_4_ and P2O_4_ tetra­hedra gives layers perpendicular to [010] (Fig. 3 ▸), which, in turn, are linked into the three-dimensional framework by sharing corners with phosphate tetra­hedra. This framework accommodates two types of channels extending parallel to [001] in which the Na^+^ cations are located (Fig. 4 ▸).

The mean <*M*1—O> distance of 2.329 Å is between those of 2.23 and 2.42 Å predicted by the sums of the ionic radii (Shannon, 1976 ▸) for Mn^2+^ and Na^+^ cations in an octa­hedral environment. The mean <*M*2—O> distance of 2.150 Å is between the mean distance of 2.142 Å observed for In^3+^ in an octa­hedral environment in NaCuIn(PO_4_)_2_ (Benhsina *et al.*, 2020 ▸) and 2.238 Å for Mn^2+^ in the same coordination in K_0.53_Mn_2.37_Fe_1.24_(PO_4_)_3_ (Hidouri & Ben Amara, 2011 ▸). The PO_4_ tetra­hedra show a slight distortion, as indicated by the range of P—O bond lengths [1.538 (2)–1.550 (2) Å for P1O_4_ and 1.520 (3)–1.566 (2) Å for P2O_4_], with mean bond lengths of <P1—O> = 1.544 (2) Å and <P2—O> = 1.546 (2) Å, consistent with 1.537 Å as calculated by Baur (1974 ▸) for the orthophosphate group. The coordination spheres of the two crystallographically distinct Na sites (Fig. 1 ▸) in the channels were defined under the assumption of a maximum Na—O distance *L*_max_ = 3.13 Å, suggested by Donnay & Allmann (1970 ▸). The environment around Na1 consists of seven O atoms with distances varying from 2.35 (3) to 2.99 (3) Å, and Na2 is bound to eight O atoms with distances in the range 2.510 (3)–2.928 (6) Å.

The refined structure model is confirmed by (i) the bond-valence method (Brown & Altermatt, 1985 ▸; Brown, 2002 ▸) and (ii) the charge-distribution (Chardi) method (Nespolo, 2015 ▸, 2016 ▸). The Chardi method is a development of Pauling’s concept of bond strength (Pauling, 1929 ▸). Instead of the empirical parameters used in the bond-valence approach, it exploits the experimental bond lengths deduced from the structural study to compute a non-integer coordination number (effective coordination number = ECoN) around a PC atom (atom placed at the center of a polyhedron, *q* > 0), which is coordinated by V atoms (atoms located at the vertices; *q* < 0); *q* is the formal oxidation number. ECoN takes into account not only the number of V atoms around a given PC atom, but also their weight in terms of relative distances. Calculated charges *Q*(*i*) and valences *V*(*i*) are in good agreement with the formal oxidation number (*q*) multiplied by occupancy rates. The dispersion factor MAPD, 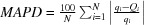, which measures the mean absolute percentage deviation, is 1% for the calculated cationic charges. The variation of the ECoN value with respect to the traditional coordination number indicates the degree of distortion. The results of the two validation models are compiled in Table 1 ▸.

## Synthesis and crystallization   

Commercially available NaNO_3_, Mn(NO_3_)_2_·6H_2_O, In_2_O_3_, MoO_3_ and (NH_4_)_2_HPO_4_ were mixed in stoichiometric ratios of 2:1:1:1:2 and dissolved in aqueous nitric acid. The resulting solution was then evaporated by heating at 353 K. The obtained dry residue was ground in an agate mortar, and then heated increasingly in an open platinum crucible up to 873 K. The sample was then reground and mixed with sodium dimolybdate Na_2_Mo_2_O_7_ in the molar ratio P:Mo = 2:1. The mixture was heated for 1 h at 1243 K to give a melt that was subsequently cooled down to room temperature at a rate of 10 K h^−1^. Brown hexa­gonally shaped crystals were obtained by washing the final product with hot water in order to dissolve the flux.

## Refinement   

Crystal data, data collection and structure refinement details are summarized in Table 2 ▸. The bond lengths involving *M*1—O and *M*2—O are those between the mean Na—O and Mn—O and the mean In—O and Mn—O bond lengths, respectively. We used EADP, EXYZ and SUMP constraints within *SHELXL2018*/3 (Sheldrick, 2015 ▸) for the mixed-occupied *M*1 [refined ratio Mn:Na = 0.5438 (14):0.4562 (14)] and *M*2 [refined ratio In:Mn = 0.8443 (5):0.1557 (5)] sites. Na2 shows an occupancy of 0.7676 (17), and free refinement of the occupancy of Na1 resulted in a value very close to 0.5. For the final refinement, this value was fixed at 0.5, and all other occupancies were refined to ensure electrical neutrality of the compound. The remaining maximum and minimum electron densities are located 0.74 Å from P2 and 1.07 Å from O24, respectively.

## Supplementary Material

Crystal structure: contains datablock(s) global, I. DOI: 10.1107/S2056989020010191/wm5566sup1.cif

Structure factors: contains datablock(s) I. DOI: 10.1107/S2056989020010191/wm5566Isup2.hkl

CHARDI and BVS analysis of cation polyhedra in Na2.22Mn0.87In1.68(PO4)3. DOI: 10.1107/S2056989020010191/wm5566sup3.docx

CCDC reference: 2018562

Additional supporting information:  crystallographic information; 3D view; checkCIF report

## Figures and Tables

**Table 1:** CHARDI and BVS analysis of cations in Na_2.22_Mn_0.87_In_1.68_(PO_4_)_3_ *M*1 = Mn/Na, *M*2 = Mn/In, *q* = formal oxidation number, sof(*i*) = site-occupation factor, *Q*(*i*) = calculated charges, CN = coordination number, ECoN = number of effective coordination, *d*_ar_ = arithmetic average distance to oxygen atoms and *d*_med_ = weighted average distance to oxygen atoms.

Cation	*q*.sof(i)	*Q*(*i*)	*V*(*i*).sof(*i*)	CN(*i*)	ECoN(*i*)	*d*_average_	*d*_med_
Na1	0.50	0.50	0.542	7	5.56	2.550	2.428
Na2	0.77	0.75	0.716	8	6.70	2.738	2.653
*M*1	1.54	1.56	1.421	6	6.00	2.329	2.330
*M*2	2.84	2.84	2.696	6	5.87	2.150	2.141
P1	5.00	4.92	4.873	4	4.00	1.544	1.544
P2	5.00	5.05	4.844	4	3.98	1.546	1.545

**Table 2:** Experimental details

Crystal data	
Chemical formula	Na_2.22_Mn_0.87_In_1.68_(PO_4_)_3_
*M*_r_	575.82
Crystal system, space group	Monoclinic, *C*2/*c*
Temperature (K)	293
*a*, *b*, *c* (Å)	12.412 (2), 12.855 (2), 6.599 (1)
β (°)	114.727 (2)
*V* (Å^3^)	956.4 (3)
*Z*	4
Radiation type	Mo *K*α
μ (mm^−1^)	5.81
Crystal size (mm)	0.29 × 0.17 × 0.11

Data collection	
Diffractometer	Nonius Kappa CCD
Absorption correction	Part of the refinement model (Δ*F*) (Parkin *et al.*, 1995 ▸)
*T*_min_, *T*_max_	0.178, 0.222
No. of measured, independent and observed [*I* > 2σ(*I*)] reflections	1183, 1183, 1172
*R*_int_	0.034
(sin θ/λ)_max_ (Å^−1^)	0.680

Refinement	
*R*[*F* ^2^ > 2σ(*F* ^2^)], *wR*(*F* ^2^), *S*	0.021, 0.054, 1.31
No. of reflections	1183
No. of parameters	102
No. of restraints	3
Δρ_max_, Δρ_min_ (e Å^−3^)	0.73, −0.83

Computer programs: *KappaCCD Server Software* (Nonius, 1997 ▸), *HKL* *SCALEPACK* and *DENZO* (Otwinovski & Minor, 1997 ▸), *SIR92* (Altomare *et al.*, 1993 ▸), *SHELXL2018/3 (Sheldrick, 2015 ▸)*, *ORTEP-3 for Windows* and *WinGX* (Farrugia, 2012 ▸) and *DIAMOND* (Brandenburg, 1999 ▸).

## supplementary crystallographic information

### Crystal data

**Table:**

Na_2.22_Mn_0.87_In_1.68_(PO_4_)_3_	*F*(000) = 1100
*M**_r_* = 575.82	*D*_x_ = 4.115 Mg m^−^^3^
Monoclinic, *C*2/*c*	Mo *K*α radiation, λ = 0.71073 Å
Hall symbol: -C 2yc	Cell parameters from 25 reflections
*a* = 12.412 (2) Å	θ = 3.2–28.9°
*b* = 12.855 (2) Å	µ = 5.81 mm^−^^1^
*c* = 6.599 (1) Å	*T* = 293 K
β = 114.727 (2)°	Prism, brown
*V* = 956.4 (3) Å^3^	0.29 × 0.17 × 0.11 mm
*Z* = 4	

### Data collection

**Table:**

Nonius Kappa CCD diffractometer	1172 reflections with *I* > 2σ(*I*)
Graphite monochromator	*R*_int_ = 0.034
non–profiled ω/2τ scans	θ_max_ = 28.9°, θ_min_ = 2.4°
Absorption correction: part of the refinement model (Δ*F*) (Parkin *et al.*, 1995)	*h* = −16→14
*T*_min_ = 0.178, *T*_max_ = 0.222	*k* = 0→16
1183 measured reflections	*l* = 0→8
1183 independent reflections	

### Refinement

**Table:**

Refinement on *F*^2^	3 restraints
Least-squares matrix: full	Primary atom site location: structure-invariant direct methods
*R*[*F*^2^ > 2σ(*F*^2^)] = 0.021	*w* = 1/[σ^2^(*F*_o_^2^) + (0.0135*P*)^2^ + 7.1094*P*] where *P* = (*F*_o_^2^ + 2*F*_c_^2^)/3
*wR*(*F*^2^) = 0.054	(Δ/σ)_max_ = 0.023
*S* = 1.31	Δρ_max_ = 0.73 e Å^−^^3^
1183 reflections	Δρ_min_ = −0.83 e Å^−^^3^
102 parameters	

### Special details

**Table:**

Geometry. All esds (except the esd in the dihedral angle between two l.s. planes) are estimated using the full covariance matrix. The cell esds are taken into account individually in the estimation of esds in distances, angles and torsion angles; correlations between esds in cell parameters are only used when they are defined by crystal symmetry. An approximate (isotropic) treatment of cell esds is used for estimating esds involving l.s. planes.

### Fractional atomic coordinates and isotropic or equivalent isotropic displacement parameters (Å^2^)

**Table:**

	*x*	*y*	*z*	*U*_iso_*/*U*_eq_	Occ. (<1)
Na1	0.492 (3)	0.003 (3)	0.013 (5)	0.017 (2)	0.5
Na2	0	−0.0079 (4)	0.75	0.0586 (13)	0.7676 (17)
Mn	0.5	0.22924 (8)	0.25	0.0128 (2)	0.5438 (14)
Na	0.5	0.22924 (8)	0.25	0.0128 (2)	0.4562 (14)
In	0.22327 (2)	0.15486 (2)	0.64458 (4)	0.00781 (9)	0.8443 (5)
Mn1	0.22327 (2)	0.15486 (2)	0.64458 (4)	0.00781 (9)	0.1557 (5)
P1	0.5	0.21702 (9)	0.75	0.0075 (2)	
O11	0.5471 (2)	0.2867 (2)	0.9611 (4)	0.0138 (5)	
O12	0.4072 (2)	0.14288 (19)	0.7683 (4)	0.0140 (5)	
P2	0.23767 (7)	0.10662 (6)	1.13105 (13)	0.00841 (17)	
O21	0.1736 (3)	0.0029 (2)	1.1238 (4)	0.0172 (5)	
O22	0.2293 (2)	0.17542 (19)	1.3198 (4)	0.0112 (5)	
O23	0.1663 (2)	0.16388 (19)	0.9065 (4)	0.0139 (5)	
O24	0.3668 (2)	0.0921 (2)	1.1731 (5)	0.0206 (6)	

### Atomic displacement parameters (Å^2^)

**Table:**

	*U*^11^	*U*^22^	*U*^33^	*U*^12^	*U*^13^	*U*^23^
Na1	0.023 (7)	0.013 (3)	0.012 (5)	0.004 (4)	0.005 (3)	−0.002 (3)
Na2	0.027 (2)	0.073 (3)	0.055 (3)	0	−0.0025 (19)	0
Mn	0.0146 (5)	0.0121 (5)	0.0141 (5)	0	0.0083 (4)	0
Na	0.0146 (5)	0.0121 (5)	0.0141 (5)	0	0.0083 (4)	0
In	0.00802 (13)	0.00782 (13)	0.00813 (14)	0.00050 (8)	0.00390 (10)	0.00093 (8)
Mn1	0.00802 (13)	0.00782 (13)	0.00813 (14)	0.00050 (8)	0.00390 (10)	0.00093 (8)
P1	0.0062 (5)	0.0092 (5)	0.0055 (5)	0	0.0008 (4)	0
O11	0.0097 (11)	0.0174 (12)	0.0121 (11)	0.0005 (9)	0.0024 (9)	−0.0062 (9)
O12	0.0083 (11)	0.0139 (12)	0.0184 (13)	−0.0007 (9)	0.0042 (10)	0.0045 (9)
P2	0.0131 (4)	0.0069 (4)	0.0063 (4)	0.0012 (3)	0.0051 (3)	0.0005 (3)
O21	0.0287 (14)	0.0080 (11)	0.0163 (13)	0.0006 (10)	0.0108 (11)	0.0008 (9)
O22	0.0154 (12)	0.0105 (11)	0.0081 (11)	−0.0003 (9)	0.0053 (9)	−0.0005 (9)
O23	0.0232 (13)	0.0120 (12)	0.0079 (11)	0.0027 (9)	0.0079 (10)	0.0021 (9)
O24	0.0201 (14)	0.0249 (14)	0.0206 (14)	0.0084 (11)	0.0124 (11)	0.0071 (11)

### Geometric parameters (Å, º)

**Table:**

Na1—O12^i^	2.35 (3)	Mn—O23^xi^	2.330 (3)
Na1—O12^ii^	2.38 (3)	Mn—O11^iii^	2.335 (3)
Na1—O24^iii^	2.38 (3)	Mn—O11^i^	2.335 (3)
Na1—O24^iv^	2.45 (3)	In—O12	2.084 (2)
Na1—O24^i^	2.489 (18)	In—O21^v^	2.107 (3)
Na1—O24^ii^	2.811 (17)	In—O23	2.127 (3)
Na1—O12^v^	2.99 (3)	In—O11^xii^	2.144 (2)
Na2—O21	2.510 (3)	In—O22^i^	2.192 (2)
Na2—O21^vi^	2.510 (3)	In—O22^xiii^	2.246 (2)
Na2—O21^v^	2.616 (3)	P1—O12	1.538 (2)
Na2—O21^vii^	2.616 (3)	P1—O12^iii^	1.538 (2)
Na2—O23^vi^	2.901 (5)	P1—O11^iii^	1.550 (2)
Na2—O23	2.901 (5)	P1—O11	1.550 (2)
Na2—O11^viii^	2.928 (6)	P2—O24	1.520 (3)
Na2—O11^ix^	2.928 (6)	P2—O21	1.543 (3)
Mn—O24^i^	2.323 (3)	P2—O23	1.557 (3)
Mn—O24^iii^	2.323 (3)	P2—O22	1.566 (2)
Mn—O23^x^	2.330 (3)		
			
O12^i^—Na1—O12^ii^	172.0 (8)	O21^vii^—Na2—O11^ix^	84.12 (12)
O12^i^—Na1—O24^iii^	100.6 (13)	O23^vi^—Na2—O11^ix^	145.72 (9)
O12^ii^—Na1—O24^iii^	80.8 (10)	O23—Na2—O11^ix^	123.11 (7)
Na1^xiv^—Na1—O24^iv^	73 (10)	O11^viii^—Na2—O11^ix^	51.21 (13)
O12^i^—Na1—O24^iv^	79.9 (10)	O24^i^—Mn—O24^iii^	81.29 (13)
O12^ii^—Na1—O24^iv^	97.6 (12)	O24^i^—Mn—O23^x^	164.09 (10)
O24^iii^—Na1—O24^iv^	172.4 (9)	O24^iii^—Mn—O23^x^	86.16 (9)
O12^i^—Na1—O24^i^	76.2 (8)	O24^i^—Mn—O23^xi^	86.16 (9)
O12^ii^—Na1—O24^i^	111.7 (10)	O24^iii^—Mn—O23^xi^	164.09 (10)
O24^iii^—Na1—O24^i^	76.9 (7)	O23^x^—Mn—O23^xi^	107.74 (13)
O24^iv^—Na1—O24^i^	110.6 (11)	O24^i^—Mn—O11^iii^	91.16 (9)
O12^i^—Na1—O24^ii^	102.3 (8)	O24^iii^—Mn—O11^iii^	117.37 (9)
O12^ii^—Na1—O24^ii^	69.7 (6)	O23^x^—Mn—O11^iii^	85.95 (9)
O24^iii^—Na1—O24^ii^	102.7 (9)	O23^xi^—Mn—O11^iii^	72.40 (9)
O24^iv^—Na1—O24^ii^	69.8 (6)	O24^i^—Mn—O11^i^	117.37 (9)
O24^i^—Na1—O24^ii^	178.4 (15)	O24^iii^—Mn—O11^i^	91.16 (9)
O12^i^—Na1—O12^v^	135.0 (10)	O23^x^—Mn—O11^i^	72.40 (9)
O12^ii^—Na1—O12^v^	51.9 (6)	O23^xi^—Mn—O11^i^	85.95 (9)
O24^iii^—Na1—O12^v^	96.7 (9)	O11^iii^—Mn—O11^i^	143.12 (14)
O24^iv^—Na1—O12^v^	88.1 (10)	O12—In—O21^v^	101.37 (10)
O24^i^—Na1—O12^v^	67.8 (5)	O12—In—O23	111.59 (10)
O24^ii^—Na1—O12^v^	113.8 (11)	O21^v^—In—O23	85.28 (10)
O21—Na2—O21^vi^	173.7 (3)	O12—In—O11^xii^	159.80 (10)
O21—Na2—O21^v^	80.14 (8)	O21^v^—In—O11^xii^	95.68 (10)
O21^vi^—Na2—O21^v^	99.71 (8)	O23—In—O11^xii^	80.34 (10)
O21—Na2—O21^vii^	99.71 (8)	O12—In—O22^i^	84.96 (10)
O21^vi^—Na2—O21^vii^	80.14 (8)	O21^v^—In—O22^i^	100.43 (10)
O21^v^—Na2—O21^vii^	177.2 (3)	O23—In—O22^i^	161.27 (10)
O21—Na2—O23^vi^	119.88 (19)	O11^xii^—In—O22^i^	81.35 (9)
O21^vi^—Na2—O23^vi^	54.39 (10)	O12—In—O22^xiii^	80.47 (9)
O21^v^—Na2—O23^vi^	115.22 (16)	O21^v^—In—O22^xiii^	176.57 (10)
O21^vii^—Na2—O23^vi^	62.40 (10)	O23—In—O22^xiii^	91.36 (9)
O21—Na2—O23	54.39 (10)	O11^xii^—In—O22^xiii^	83.08 (9)
O21^vi^—Na2—O23	119.88 (19)	O22^i^—In—O22^xiii^	82.57 (9)
O21^v^—Na2—O23	62.40 (10)	O12—P1—O12^iii^	103.4 (2)
O21^vii^—Na2—O23	115.22 (16)	O12—P1—O11^iii^	114.56 (13)
O23^vi^—Na2—O23	80.89 (17)	O12^iii^—P1—O11^iii^	107.48 (14)
O21—Na2—O11^viii^	115.83 (18)	O12—P1—O11	107.48 (14)
O21^vi^—Na2—O11^viii^	70.36 (11)	O12^iii^—P1—O11	114.56 (13)
O21^v^—Na2—O11^viii^	84.12 (12)	O11^iii^—P1—O11	109.4 (2)
O21^vii^—Na2—O11^viii^	98.43 (14)	O24—P2—O21	112.98 (16)
O23^vi^—Na2—O11^viii^	123.11 (7)	O24—P2—O23	111.62 (15)
O23—Na2—O11^viii^	145.71 (9)	O21—P2—O23	107.34 (15)
O21—Na2—O11^ix^	70.36 (11)	O24—P2—O22	109.88 (15)
O21^vi^—Na2—O11^ix^	115.83 (18)	O21—P2—O22	107.94 (14)
O21^v^—Na2—O11^ix^	98.43 (14)	O23—P2—O22	106.81 (14)

Symmetry codes: (i) *x*, *y*, *z*−1; (ii) −*x*+1, −*y*, −*z*+1; (iii) −*x*+1, *y*, −*z*+3/2; (iv) *x*, −*y*, *z*−3/2; (v) *x*, −*y*, *z*−1/2; (vi) −*x*, *y*, −*z*+3/2; (vii) −*x*, −*y*, −*z*+2; (viii) −*x*+1/2, *y*−1/2, −*z*+3/2; (ix) *x*−1/2, *y*−1/2, *z*; (x) *x*+1/2, −*y*+1/2, *z*−1/2; (xi) −*x*+1/2, −*y*+1/2, −*z*+1; (xii) *x*−1/2, −*y*+1/2, *z*−1/2; (xiii) −*x*+1/2, −*y*+1/2, −*z*+2; (xiv) −*x*+1, −*y*, −*z*.
