# Supplementary material for: Synthesis, crystal structure and charge-distribution validation of a new alluaudite-type phosphate, Na2.22Mn0.87In1.68(PO4)3
Source: Acta Crystallogr E Crystallogr Commun. 2020 Jul 31;76(Pt 8):1369–72. doi: 10.1107/S2056989020010191 (PMC7405582; doi:10.1107/S2056989020010191)
Supplement: Supplementary file 3 [file e-76-01369-sup3.docx]

**Table 2:** CHARDI and BVS analysis of cation polyhedra in Na_2.22_Mn_0.87_In_1.68_(PO_4_)_3_

| **Cation** | **q.sof(i)** | **Q(i)** | **V(i).sof(i)** | **CN(i)** | **ECoN(i)** | **d_ar_** | **d_med_** |
| --- | --- | --- | --- | --- | --- | --- | --- |
| Na1 | 0.50 | 0.5 | 0.542 | 7 | 5.56 | 2.428 | 2.428 |
| Na2 | 0.77 | 0.75 | 0.716 | 8 | 6.70 | 2.653 | 2.653 |
| M(1) | 1.54 | 1.56 | 1.421 | 6 | 6.00 | 2.330 | 2.330 |
| M(2) | 2.84 | 2.84 | 2.696 | 6 | 5.87 | 2.141 | 2.141 |
| P1 | 5.00 | 4.92 | 4.873 | 4 | 4.00 | 1.544 | 1.544 |
| P2 | 5.00 | 5.05 | 4.844 | 4 | 3.98 | 1.545 | 1.545 |

Notes: M(1)= Mn/Na, M(2)=Mn/In, q(i) = formal oxidation number, sof(i) = site-occupation factor, Q(i) = calculated charges, CN = coordination number, ECoN = number of effective coordination, $MAPD= 100/N\sum_{i}^{N} \left| q_{i}-\frac{Q_{i}}{q_{i}} \right|$, d_ar_=arithmetic average distance and d_med_=weighted average distance.
